# Long-term care provision, hospital bed blocking, and discharge destination for hip fracture and stroke patients

**DOI:** 10.1007/s10754-017-9214-z

**Published:** 2017-02-28

**Authors:** James Gaughan, Hugh Gravelle, Rita Santos, Luigi Siciliani

**Affiliations:** 1 Centre for Health Economics, University of York, York, UK; 2 Department of Economics and Related Studies, University of York, York, UK

**Keywords:** Hospitals, Length of stay, Long-term care, Care homes, Bed blocking, Substitution, I11

## Abstract

We examine the relationship between long-term care supply (care home beds and prices) and (i) the probability of being discharged to a care home and (ii) length of stay in hospital for patients admitted to hospital for hip fracture or stroke. Using patient level data from all English hospitals and allowing for a rich set of demographic and clinical factors, we find no association between discharge destination and long-term care beds supply or prices. We do, however, find evidence of bed blocking: hospital length of stay for hip fracture patients discharged to a care home is shorter in areas with more long-term care beds and lower prices. Length of stay is over 30% shorter in areas in the highest quintile of care home beds supply compared to those in the lowest quintile.

## Introduction

The provision of health care and long-term care for the elderly is a consistent focus of policy makers in the U.K. and other OECD countries (Department of Health 2001, 2011; Glendinning 2003; OECD 2011; Wanless 2006). Around 10% of individuals over 75 years old used both health and long-term care in 2006/2007 in England (Bardsley et al. 2012). Long- term care has costs and outcome consequences on health care and vice versa (Fernandez and Forder 2008; Forder 2009; Vetter 2003). In England, acute hospital care and long-term care are organised and funded separately and differently.

There is long standing concern over coordination for patients requiring health and long-term care, in particular the delayed discharge of patients from hospital (Baumann et al. 2007; Bryan et al. 2006; House of Commons 2003; National Audit Office 2000).To improve integration policy makers need information about the effects of provision of one type of care on the other. In this paper we examine two questions where there is little quantitative evidence: the extent to which accessibility of long-term care affects the length of stay in hospital and the probability of a patient being discharged back to their homes rather than to a care home.

We focus on patients who suffer a hip fracture or stroke. The conditions are selected for their high incidence, impairing effects on patients, and the consequent need for long-term care for the elderly (Kasteridis et al. 2015; de Maijer et al. 2011). The British Orthopaedics association reports there were around 70,000 hip fractures in the U.K. in 2007, including many older patients with complex clinical needs. The incidence is expected to rise (British Orthopaedic Association 2007). Further, 10–20% of hip fracture patients admitted to hospital from their own home ultimately utilise some institutional care (National Clinical Guidelines Centre 2011). The national stroke strategy published by the Department of Health in 2007 reports that there are approximately 110,000 strokes each year in England and that 75% of these occur among people aged 65 and over. Stroke is the single largest cause of disability, with a third of people suffering from a stroke having a long-term disability (Department of Health 2007; National Audit Office 2005). Estimates of the proportion of care home residents who have had a stroke vary between 25 and 45% (Care Quality Commission 2011). Hip fracture and stroke patients are thus a policy concern (Department of Health 2001) and have been the focus of past research in England (Bond et al. 2000). Both require immediate hospital care and longer term rehabilitation. Such rehabilitation could take place in hospital but also at the patient’s home or in a long-term care facility.

Higher care home bed supply, at given prices, implies a shorter waiting time for a bed to become available and may thus increase the probability of discharge to a care home. It may also imply a shorter hospital length of stay since patients will have shorter waits for a place in a care home. Higher prices will reduce probability of the patient opting for a care home as opposed to returning to their own home and may induce patients to search for longer, thereby increasing hospital length of stay.

We examine two questions. First, we investigate whether access to long-term care in nursing and residential homes (as measured by beds and prices) influences the probability that patients who are admitted to hospital from their home are discharged to a care home. Second, we investigate the bed blocking hypothesis that the supply of long-term care influences length of stay in hospital.

### Institutional background

Emergency hospital care in England is predominantly provided by the National Health Service (NHS) through 166 acute public hospitals, known as Trusts. NHS care is funded by general taxation, with 152 local health authorities (Primary Care Trusts, PCTs) receiving capitated budgets from the Department of Health from which they pay for hospital care provided to their populations. Patients do not pay for hospital care provided by NHS hospitals.

Long-term care is provided by over 18,000 care homes^1^ (Laing and Buisson 2010). In 2014 about 74% of care home beds were provided by for-profit firms, 17% by voluntary organisations and 8% by Local Authorities (LAs) (Jarret 2016).^2^ Providers are generally small and the supply and price of care home beds is largely driven by local demand, the cost of provision, and by competition (Forder and Fernandez 2012; Allan and Forder 2015).

Around 60% of care home users (Forder 2007) are self-funders and pay at least in part for their long-term care. Those with low wealth are subsidised by their LA. The price charged for subsidised patients is lower than for self-funders because of the bargaining power of the LAs (OFT 2005). This gives a financial incentive to care homes to give priority to self-funders, within the constraints of any locally negotiated contract with LA commissioners. Despite potentially considerable out-of-pocket expenses associated with long-term care, the proportion of individuals covered by private long-term care insurance in England is very small, with just under 22,000 people holding such insurance in 2008 (Comas-Herrera et al. 2009).

The majority of residents in care homes are long stay. Care homes also provide some rehabilitation services. Steventon and Roberts (2012) found that 39% of LA funded admissions to care homes in three English LAs in 2005/2006 were short stay but that the median length of stay was 18 months.

### Previous literature

Previous studies investigating discharge destination of hospital patients find that age, gender and living arrangements are key drivers of discharge to a care home as opposed to their own home. Other factors include comorbidities (Aharonoff et al. 2004; Gilbert et al. 2010), ethnicity (Aharonoff et al. 2004; Ellis and Trent 2001), urbanisation (Gilbert et al. 2010) and income deprivation (Gilbert et al. 2010; Picone et al. 2003). Patients discharged to a care home are also likely to have a longer stay in hospital (Castelli et al. 2015; Wong et al. 2010).

Picone et al. (2003) investigates the determinants of hospital length of stay and discharge destination of 4608 U.S. Medicare patients following hip fracture, stroke or heart attack. They show that potential supply of informal care (being married and number of children) increases the likelihood of being discharged home. They also find that supply of institutional care (concentration of skilled nursing home or rehabilitation hospital beds) is associated with shorter length of stay. The only English study of discharge destination is Bond et al. (2000) who examine the discharge destination of 440 stroke and 572 hip fracture patients in six English NHS hospitals. They find that the probability of being discharged to a care home increases with the supply of care home beds. In line with these studies, we find a negative association between care home beds supply and length of stay in hospitals, and a positive but insignificant association between care home beds supply and the probability of being discharged to a care home.

Although there is an extensive literature on the substitution between informal and formal long-term care (Bolin et al. 2008; Bonsang 2009; Grabowski et al. 2012; Van Houtven and Norton 2004), there is limited evidence on the effect of care homes supply on health care utilisation, i.e. on the substitution between long-term social care and hospital care. Fernandez and Forder (2008) find that LAs with more home help hours and nursing and residential care beds have a lower rate of hospital delayed discharges and lower emergency readmission rates. Forder (2009) uses small area data on 8000 census areas in England and finds that a £1 increase in spending on care homes reduces hospital expenditure by £0.35. Gaughan et al. (2015) use LA level data on all types of hospital patients and find that greater supply of care home beds in the LA is associated with a reduction in delayed discharge from hospital.

Holmås et al. (2010) investigate the effect of fining owners of long-term care institutions which prolong length of stay at hospitals in Norway. Surprisingly, hospital length of stay is longer when the fines are used, which they interpret as an example of monetary incentives crowding out intrinsic motivation. Holmås et al. (2013) find that greater expenditure on long-term care by Norwegian local authorities reduces both overall length of hospital stay and stay in hospital when medically ready for discharge.

Our study contributes to the literature on the relationship between long-term care and health care by using a large and rich individual patient level data set for two disabling and high incidence conditions so that we can control more precisely for patient diagnoses, socio-economic characteristics and hospital policies than previous English area level studies.

## Data and methods

### Sample

A detailed account of the data set construction is in the Data Appendix. We use cross-section administrative data from Hospital Episodes Statistics (HES). Our sample is all patients aged 65 or over, resident in England, treated in NHS hospitals, admitted from home as an emergency with a primary diagnosis of hip fracture or stroke, and discharged in the financial year April 2008–March 2009. We exclude patients who are admitted from a care home since they are very likely to return to the same care home. We analyse the two samples (hip fracture and stroke) separately. While coordination between acute and long-term care services is important for patients with either condition, the characteristics and needs of the two patient groups differ. As well as being treated in different hospital departments, which may have different policies about discharge, stroke is a potentially more severe condition and with a more varied prognosis.

Patients who die in hospital, are discharged to a penal institution or to a secure psychiatric unit, have incomplete spells, or for whom final discharge destination is not known, are excluded from the analysis. We also exclude patients at Hospital Trusts with less than 10 hip fracture and stroke patients per year. The estimation samples are 21,959 hip fracture patients and 33,101 stroke patients.

### Dependent variables

Our dependent variables are the patient’s hospital length of stay and whether the patient is discharged to a long-term care institution as opposed to returning home following hospitalisation. Patient length of stay is the number of days between admission and discharge from hospital at the end of their spell, allowing for patients to be transferred between hospitals during their spell.

### Patient characteristics

For each patient we have age, gender, number of diagnoses and procedures, day of discharge and whether the patient is transferred to a different provider during their hospital spell. HES diagnostic fields are used to construct three co-morbidity dummy variables based on the Charlson index (Charlson et al. 1987). We distinguish between (i) no Charlson co-morbidities, (ii) a single non-severe co-morbidity, (iii) at least one severe or at least two non-severe co-morbidities. Since stroke is one of the Charlson co-morbidities we exclude it when constructing these variables for stroke patients.

We also distinguish between different types of hip fracture and stroke. The major types of hip fracture relate to the location of the fracture. The categories are femoral neck, peritrochanteric, and subtrochanteric, where the trochanters refer to protrusions of bone just below the ball of the hip. Prognosis and length of stay varies by type of fracture (Butler et al. 2009; Clague et al. 2002). The most critical distinction for stroke patients is between strokes due to an infarction, where a vessel supplying blood to the brain is blocked and those due to a haemorrhage, where there is bleeding in or around the brain. Although patient outcome is driven by stroke severity as well as by type of stroke (Jørgensen et al. 1995) haemorrhagic strokes generally lead to worse outcomes in terms of disability and mortality.

### Formal long-term care

We have data on the number of registered beds and prices of different types of rooms for all providers in England whose main client group is patients aged 65 or over or with dementia (Laing and Buisson 2010). We include these two categories of provider since they match with the age restrictions of our sample and it is these groups of patients most likely to require care in a care home following hip fracture or stroke. Care homes specialising in dementia are likely to cater to patients in our group given that its prevalence increases rapidly with age. Moreover, having a stroke might cause dementia and conversely a patient suffering with dementia might have a hip fracture from a fall.

While stroke is a more severe condition, the Stroke Association suggests that a patient can be accommodated in a residential home depending on need (Stroke Association 2013). There are almost no specialist stroke care homes and at least 25% of patients in care homes have had a stroke (Care Quality Commission 2011). There is a small amount of specialist rehabilitation in the care home sector but these providers do not cater specifically for care following stroke or hip fracture.

We compute the number of beds and average prices in residential and nursing homes within a 10 km radius of the centroid of each patient’s small area of residence [(Lower Super Output Area (LSOA)]. LSOAs are small geographic areas with an average population of 1500 in 2001. There are 32,482 LSOAs in England served by 166 acute hospital trusts. We also have a measure of average care home quality based on ratings by the sector regulator [the Care Quality Commission (CQC)].

To allow for differences in population we use the mid-2008 population of retirement age (60 years and over for women; 65 years and over for men) living in LSOAs whose centroids are within 10 km of each patient’s LSOA centroid.

### Socioeconomic characteristics of patient areas of residence

Information on socioeconomic characteristics is not available at the individual level in HES. We attribute socioeconomic variables from the 2001 Census and the 2004 Index of Multiple Deprivation to patients by their LSOA of residence. The variables include the proportions of non-white residents, households with a single pensioner, and those reporting self-assessed health as “not good” from the three categories (good, fairly good and not good). The proportion of single pensioner households provides some adjustment for availability of informal care as much of this care is provided by a spouse or other relative living with the patient. We measure income deprivation as the proportion of the LSOA’s population aged over 60 who are claiming income support, income related job seekers allowance, or pension credit guarantee. LSOAs are classified as urban, town or village. We include a dummy variable for patients resident in a London LSOA to allow for peculiarities of health and long-term care provision in the capital. Because of lack of information on acute care in Scotland and Wales, we include dummy variables indicating whether a patient’s LSOA is within 10 km of the English border with Scotland or Wales.

### Methods

We use models with the same explanatories for both patient discharge destination and patient length of stay except for using day of discharge in modelling length of stay. We estimate linear probability models for a patient being discharged to a care home as opposed to the patient’s home, separately for hip fracture and stroke. The model is

1 $$\begin{aligned} y_{ij} =\beta _1 m_{ij} +\beta _2 x_{ij} +\beta _3 B_{ij} +\beta _4 P_{ij} +h_j +\varepsilon _{ij} \end{aligned}$$

where $y_{ij}$ is an indicator variable equal to one if the patients *i* in hospital *j* is discharged to a care home and zero if discharged to their own home. $m_{ij}$ is a vector of patient morbidity variables and $x_{ij}$ is a vector of patient demographic and socio-economic characteristics. $B_{ij}$ is care home beds supply in the 10 km area around the centroid of the patient’s LSOA of residence, and $P_{ij}$ is average care home prices in the same area. Notice that care home beds and prices vary across patients treated within the same hospital since each hospital will draw patients from many different LSOAs. We allow for non-linear effects by measuring price and beds supply as indicators for the quintiles of the national distribution of these variables across all LSOAs. $h_{j}$ is a hospital effect. Except where otherwise stated, we estimate all models with cluster (hospital) robust standard errors.

For the investigation of patient length of stay we use the natural logarithm of length of stay as the dependent variable in (1). We estimate separate models for patients discharged to a care home and for those discharged to their home.

To choose between fixed or random hospital effects we estimate auxiliary regressions with the same explanatory variables as (1) but with hospital effects replaced by the hospital level means of the explanatories (Mundlak 1978; Wooldridge 2010, p. 332). For all models we find that a joint Wald test rejects the null hypothesis that the coefficients on the hospital level means are zero. We therefore use the fixed effects specifications. Thus the reported coefficients on beds or prices show whether, within a given hospital, patients residing in LSOAs with a higher long-term care supply have greater length of stay and or probability of being discharged to a care home.

Care home beds and prices are measured for each patient as the sum (beds) and means (prices) for care homes within 10 km of the patient’s LSOA of residence. We argue that these are not correlated with unobservable factors affecting individual patient LoS and discharge destination. First, we control for hospital fixed effects and these will pick any variations in hospital policies which might affect length of stay or discharge destination for all patients treated in the hospital and which might be correlated with care home supply and prices if care homes locate near hospitals which have a tendency to keep patients in longer or to discharge them to care homes. We identify the effect of care home beds and prices from their variation across patients treated within the same hospital but living in different LSOAs and thus facing different beds supply and prices. Second, we have good data on individual patient morbidity which might affect length of stay and discharge destination. We also include measures of LSOA level demographics, socioeconomic characteristics, morbidity, retirement, and single pensioner households which might affect area level demand for long-term care and hence supply and prices. Third, our dependent variables are for patients with two specific conditions whilst care homes cater for a much wider set of patients so it is unlikely that care home beds supply and prices will be driven by demand from stroke and hip fracture patients.

Our models make the simplifying assumption that decisions on discharge destination and length of stay are independent. As a robustness check, we model the decisions jointly with a two-step selection model (Heckman 1979) with a probit model for discharge destination and adding the inverse Mills ratio derived from it to the linear length of stay models in the second stage. Using hospital fixed effects in the first stage probit model would yield biased estimates. Instead, we replace each explanatory variable with its hospital level mean and with patient level deviations from the mean. The hospital level means will pick up unobserved hospital effects. As an exclusion condition, we include the rate of discharge to care at the hospital level in the first stage but not in the second, arguing that the hospital rate of discharge to care will capture internal policies which affect patient probabilities of discharge to care but will not directly affect length of stay. We also run the model without this variable, relying on non-linearity for identification.

## Results

### Summary statistics

Table 1 provides descriptive statistics. Similar proportions of hip (14.5%) and stroke (13.5%) patients are discharged to a care home. The average hospital length of stay is 22 days for hip fracture and 29 days for stroke. Length of stay is shorter for patients returning to their home (20 days for hip fracture and 23 for stroke) than for those discharged to a care home (33 and 62 days). There are on average 2300–2400 care home beds within 10 km with an average price around £524 per week. The average care home quality rating is ‘good’ (a score of 3).

**Table 1:** Summary statistics

	Hip fracture				Stroke			
	Mean	Sd	Min	Max	Mean	Sd	Min	Max
*Dependent variables*								
Discharged to care home	0.145	0.352	0	1	0.135	0.342	0	1
Length of stay if discharged to care home	32.68	27.98	2	167	62.07	44.16	1	460
Length of stay if discharged home	19.95	17.90	2	168	23.38	30.13	1	394
*Patient characteristics*								
Age group: 65–74	0.166	0.372	0	1	0.299	0.458	0	1
Age group: 75–84	0.409	0.492	0	1	0.429	0.495	0	1
Age group: 85 plus	0.425	0.494	0	1	0.271	0.445	0	1
Male patient	0.223	0.416	0	1	0.466	0.499	0	1
Total diagnoses	5.713	2.919	1	39	6.234	3.511	1	32
Total procedures	2.818	1.546	0	24	2.671	1.786	0	22
Patient transferred in CIPS	0.049	0.217	0	1	0.137	0.343	0	1
Pertrochanteric fracture	0.228	0.420	0	1				
Subtrochanteric fracture	0.029	0.167	0	1				
Unspecified hip fracture	0.743	0.437	0	1				
Stroke caused by a hemorrhage					0.137	0.344	0	1
Stroke caused by an infarction					0.615	0.487	0	1
Stroke not hemorrhage or infarction					0.213	0.410	0	1
Occluded cerebral vessels no infarction					0.003	0.051	0	1
Other stroke					0.032	0.176	0	1
No Charlson comorbidities	0.493	0.500	0	1	0.515	0.500	0	1
1 minor Charlson comorbidity	0.334	0.472	0	1	0.266	0.442	0	1
$\ge$2 minor or $\ge$1 major Charlson comorbidity	0.172	0.378	0	1	0.218	0.413	0	1
Discharged on Monday	0.152	0.359	0	1	0.174	0.379	0	1
Discharged on Tuesday	0.191	0.393	0	1	0.186	0.389	0	1
Discharged on Wednesday	0.190	0.392	0	1	0.185	0.388	0	1
Discharged on Thursday	0.180	0.385	0	1	0.180	0.385	0	1
Discharged on Friday	0.209	0.406	0	1	0.216	0.411	0	1
Discharged on Saturday	0.052	0.223	0	1	0.044	0.204	0	1
Discharged on Sunday	0.017	0.131	0	1	0.015	0.122	0	1
*Socioeconomic characteristics of patient areas of residence*								
% LSOA 60+ pop on income based benefit	19.65	11.81	1.0	95.0	19.81	12.31	1.0	95.0
% LSOA pop who are non-white	6.21	11.20	0	90.45	7.09	13.00	0	94.8
% LSOA pop with good SAH	67.13	6.51	37.3	87.6	67.22	6.34	37.3	87.0
% LSOA pop with fairly good SAH	23.03	3.47	10.4	36.1	23.07	3.41	10.7	37.3
% LSOA pop with not good SAH	9.84	3.62	1.7	31.0	9.71	3.51	1.7	31.0
% single pensioner households in LSOA	16.09	6.01	0.5	51.0	15.92	6.00	0.0	51.0
Patient resident in London	0.089	0.284	0	1	0.103	0.304	0	1
LSOA within 10 km of Scottish border	0.001	0.026	0	1	0.001	0.029	0	1
LSOA within 10 km of Welsh border	0.012	0.109	0	1	0.015	0.121	0	1
Urban $>{10}\,\hbox {k}$ people	0.791	0.407	0	1	0.788	0.409	0	1
Town and fringe	0.111	0.314	0	1	0.114	0.318	0	1
Village or hamlet and isolated dwellings	0.098	0.297	0	1	0.098	0.297	0	1
Total retired population within 10 km (000s)	67.2	62.1	0.5	328.7	70.9	67.1	348	328.2
*Formal long-term care*								
Care home beds within 10 km (000 s)	2.31	1.79	0	7.81	2.41	1.92	0	7.82
Beds within 10 km/retired population	0.037	0.010	0	0.116	0.037	0.010	0	0.116
Average price within 10 km	523.21	93.05	232	971	525.25	91.49	232	961
Average care home rating within 10 km	3.03	0.17	1	4	3.03	0.17	1	4
Number of patients	21959				33101			

*CIPS* continuous in-patient spell, *LSOA* lower super output area, *SAH* self-assessed health

### Discharge destination: hip fracture

Table 2 reports the results from linear probability discharge destination models. The probability of being discharged to a care home is greater for patients who are older, female, and have more diagnoses. Compared to patients aged 65–74 years old, patients who are 75–84 and older than 84 years have 6.2 and 11.4% points higher probabilities of being discharged to a care home. Men have a 1.6% points smaller probability. An additional diagnosis or procedure increases the probability by 1.1 and 0.5% points.

**Table 2:** Determinants of discharge to care home

	Hip fracture		Stroke	
	b	p	b	p
*Patient characteristics*				
Age 75–84	0.062	0.000	0.054	0.000
Age 85 plus	0.114	0.000	0.125	0.000
Male	$-$0.016	0.005	$-$0.043	0.000
Number diagnoses	0.011	0.000	0.018	0.000
Number procedures	0.005	0.021	0.002	0.110
Patient transferred	0.005	0.793	0.029	0.025
Pertrochanteric fracture	0.000	0.938		
Subtrochanteric fracture	0.004	0.766		
Stroke caused by a hemorrhage			0.014	0.035
Stroke hemorrhage or infarction			$-$0.019	0.000
Occluded cerebral no infarction			0.009	0.776
Other stroke			$-$0.074	0.000
1 minor Charlson comorbidity	0.031	0.000	0.002	0.655
$\ge$2 minor $\ge$1 major Charlson comorbidity	0.019	0.047	$-$0.001	0.926
*Socioeconomic characteristics of patient areas of residence*				
LSOA $5\mathrm{th}$ Income deprivation quintile	$-$0.013	0.168	$-$0.012	0.050
% LSOA pop non white	0.000	0.615	0.000	0.381
% LSOA not good SAH	0.000	0.788	0.004	0.002
% LSOA single pensioner household	0.001	0.278	$-$0.001	0.080
London LSOA	$-$0.031	0.364	$-$0.027	0.371
LSOA 10 km of Scottish border	$-$0.012	0.897	$-$0.069	0.252
LSOA 10 km of Welsh border	$-$0.003	0.923	0.024	0.238
Town and fringe	$-$0.007	0.340	$-$0.003	0.560
Village, hamlet, isolated dwellings	$-$0.011	0.204	$-$0.004	0.604
Population within 10 km (100000 s)	0.013	0.578	0.011	0.449
*Formal long-term care*				
Beds within 10 km second quintile	0.009	0.396	0.002	0.790
Beds within 10 km third quintile	0.021	0.088	$-$0.012	0.238
Beds within 10 km fourth quintile	0.025	0.263	0.018	0.188
Beds within 10 km top quintile	0.041	0.197	0.014	0.512
Price within 10 km second quintile	$-$0.010	0.339	0.002	0.855
Price within 10 km third quintile	$-$0.012	0.326	0.011	0.267
Price within 10 km fourth quintile	0.006	0.739	0.019	0.127
Price within 10 km top quintile	$-$0.004	0.817	0.020	0.175
Care home ratings 10 km mean	$-$0.005	0.834	0.001	0.935
Constant	$-$0.021	0.788	$-$0.063	0.239
Hospital effects	FE		FE	
$\hbox {R}^{2}$	0.0327		0.074	
Observations	21959		33101	

Fixed effect panel data linear probability model of discharge to care home versus discharge to own home with cluster robust standard errors. The $\upchi ^{2}$ statistic for the auxiliary regression test (see “Methods” section) for the consistency of the random error specification is $\upchi ^{2}$(29)= 28.15, $p=0.5097$ for hip fracture and $\upchi ^{2}$(31)= 125.85, $p = 0.000$ for stroke

There is some, though weak, association of the probability of discharge to a care home with long-term care beds. Compared to patients in the lowest quintile of beds supply, the estimated probability of being discharged to care for patients in LSOAs in the second, third, fourth and fifth beds quintiles are 0.9, 2.1, 2.5 and 4.1% points greater. However, none of the coefficients achieves 5% statistical significance

### Discharge destination: stroke

The effects of covariates on the discharge destination of stroke patients are similar to those for hip fracture patients. Patients who are 75–84 years old and older than 84 years have 5.4 and 12.5% points higher probability of being discharged to a care home, compared to those aged 65–74. Men have a smaller probability (by 4.3% points) of being discharged to a care home.

An additional diagnosis increases the probability by 1.8% points. Transferred patients have 2.9% points higher probability. Compared to patients with cerebral infarction, the probability of discharge to care is 1.4% points higher if stroke is haemorrhagic.

The probability of stroke patients being discharged to care is not associated with the supply of care home beds, or with their price.

### Length of stay: hip fracture

Since we use the logarithm of the length of stay as the dependent variable, the coefficients in Table 3 are the proportionate changes in length of stay in days from a one unit increase in the explanatory variable. Older patients have a longer length of stay. Among patients discharged to care, those who are older than 84 years have 12% longer stays than those aged 65–74. For patients discharged home, those aged 75–84 stay 21 and those aged over 84 stay 32% longer.

**Table 3:** Determinants of length of stay: hip fracture

	Discharged to care		Discharged to home	
	b	p	b	p
*Patient characteristics*				
Age 75–84	0.065	0.128	0.209	0.000
Age 85 plus	0.120	0.007	0.315	0.000
Male	0.067	0.026	0.008	0.464
Number diagnoses	0.089	0.000	0.081	0.000
Number procedures	0.077	0.000	0.080	0.000
Patient transferred	0.855	0.000	0.870	0.000
Pertrochanteric fracture	$-$0.036	0.272	$-$0.005	0.651
Subtrochanteric fracture	0.035	0.631	0.117	0.000
1 minor Charlson comorbidity	0.009	0.769	$-$0.039	0.001
$\ge$2 minor/ $\ge$1 major Charlson comorbidity	$-$0.136	0.000	$-$0.074	0.000
Discharged on Tuesday	$-$0.087	0.037	$-$0.076	0.000
Discharged on Wednesday	$-$0.175	0.000	$-$0.082	0.000
Discharged on Thursday	$-$0.110	0.003	$-$0.086	0.000
Discharged on Friday	$-$0.141	0.000	$-$0.116	0.000
Discharged on Saturday	$-$0.154	0.006	$-$0.189	0.000
Discharged on Sunday	$-$0.197	0.057	$-$0.135	0.000
*Socioeconomic characteristics of patient areas of residence*				
LSOA $5\mathrm{th}$ income deprivation quintile	0.023	0.547	0.075	0.000
% LSOA pop non white	0.000	0.844	0.001	0.281
% LSOA not good SAH	$-$0.006	0.365	$-$0.008	0.002
% LSOA households single pensioner	$-$0.002	0.303	0.005	0.000
London LSOA	$-$0.185	0.468	$-$0.018	0.784
LSOA 10 km of Scottish border	0.448	0.000	0.099	0.079
LSOA 10 km of Welsh border	$-$0.224	0.084	$-$0.140	0.012
Town and fringe	0.009	0.849	0.000	0.989
Village, hamlet isolated dwellings	$-$0.105	0.045	$-$0.048	0.014
Population within 10 km (100000 s)	0.230	0.040	0.048	0.095
*Formal long-term care*				
Beds within 10 km second quintile	$-$0.049	0.336	$-$0.012	0.466
Beds within 10 km third quintile	$-$0.064	0.391	0.011	0.598
Beds within 10 km fourth quintile	$-$0.216	0.036	0.007	0.835
Beds within 10 km top quintile	$-$0.319	0.020	$-$0.022	0.641
Price within 10 km second quintile	$-$0.014	0.769	$-$0.030	0.178
Price within 10 km third quintile	0.006	0.925	$-$0.044	0.116
Price within 10 km fourth quintile	0.162	0.030	$-$0.031	0.342
Price within 10 km top quintile	0.175	0.050	$-$0.010	0.792
Care home ratings 10 km mean	0.127	0.182	0.030	0.428
Constant	1.977	0.000	1.782	0.000
Hospital effects	FE		FE	
$\hbox {R}^{2}$	0.305		0.311	
Observations	3175		18784	

Dependent variable: natural logarithm of length of stay. Coefficients are the proportionate change in length of stay in days from a one unit increase in the explanatory variable. Fixed effects panel data models with cluster robust standard errors. The $\upchi ^{2}$ statistic for the auxiliary regression test (see “Methods” section) for the consistency of the random error specification is $\upchi ^{2}$(35)= 115.56, $p= 0.000$ for patients discharged to care and $\upchi ^{2}$(35)= 100.87, $p = 0.000$ for those discharged to their own home

Patients discharged to care stay 6.7% longer if male, 8.9% longer with an additional diagnosis, and 7.7% with an additional procedure. Patients transferred to a different hospital have more than 80% longer stays. Surprisingly, having Charlson comorbidities reduces length of stay. Patients discharged to care who live in villages and sparsely populated areas have 11% shorter stays than those living in urban areas. The fifth most income deprived quintile of the population have 8% longer length of stay than those in less deprived areas if discharged home. Patients discharged on Monday have longer stays compared with those discharged on other days of the week, perhaps because some could not be discharged during the weekend as relevant staff were not available.

The accessibility of long-term care beds is associated with shorter lengths of stay for patients discharged to a care home: patients in LSOAs in higher quintiles of long-term care beds have shorter hospital stay than those in lower quintiles. Those in the top two quintiles have lengths of stay which are 22 and 32% shorter than those in the bottom quintile, a difference which is quantitatively large and statistically significant at 5%. There is no association between beds supply and length of stay for hip fracture patients discharged home.

There is some indication that patients in areas with higher care home prices stay longer before being discharged to a care home. Patients in the fourth and fifth quintile of the price distribution have stays which are 16% ($p = 0.030$) and 18% ($p = 0.050$) longer than those in the bottom quintile. There is no association between prices and length of stay for hip fracture patients discharged to their home.

### Length of stay: stroke

Table 4 suggests that among patients discharged home, those who are 75–84 years old and older than 84 years have greater length of stay (respectively by 16 and 33%). Older patients discharged to care have shorter stays (by 7 and 23%). Men have 15% shorter stays if discharged home. An additional procedure increases length of stay by 5% if discharged to care and 10% if discharged home. An additional diagnosis increases length of stay by 7 and 13%. Length of stay for patients transferred to another hospital is 51% greater if discharged home and 91% greater if discharged into long-term care. Patients with Charlson comorbidities have a shorter length of stay whether discharged to their own home or to care. Patients living in areas in the fifth most income deprived quintile have 4% longer length of stay if discharged home. The type of stroke also affects length of stay: compared to patients whose stroke is caused by cerebral infarction, length of stay is shorter by 50 and 67% for patients discharged to care and those discharged to home whose cause of stroke is unspecified.

**Table 4:** Determinants of length of stay: stroke

	Discharged to care		Discharged to home	
	b	p	b	p
*Patient characteristics*				
Age 75–84	$-$0.074	0.035	0.160	0.000
Age 85 plus	$-$0.225	0.000	0.327	0.000
Male	0.027	0.282	$-$0.146	0.000
Number diagnoses	0.069	0.000	0.129	0.000
Number procedures	0.052	0.000	0.099	0.000
Patient transferred	0.509	0.000	0.909	0.000
Stroke caused by a hemorrhage	$-$0.080	0.010	0.026	0.293
Stroke hemorrhage or infarction	$-$0.087	0.005	$-$0.202	0.000
Occluded cerebral vessels no infarction	0.083	0.569	$-$0.192	0.106
Other stroke	$-$0.497	0.000	$-$0.672	0.000
1 minor Charlson comorbidity	$-$0.128	0.000	$-$0.077	0.000
$\ge$2 minor or $\ge$1 major Charlson comorbidity	$-$0.207	0.000	$-$0.045	0.041
Discharged on Tuesday	$-$0.075	0.024	$-$0.156	0.000
Discharged on Wednesday	$-$0.080	0.019	$-$0.170	0.000
Discharged on Thursday	$-$0.102	0.008	$-$0.164	0.000
Discharged on Friday	$-$0.099	0.008	$-$0.313	0.000
Discharged on Saturday	$-$0.223	0.000	$-$0.423	0.000
Discharged on Sunday	$-$0.203	0.117	$-$0.675	0.000
*Socioeconomic characteristics of patient areas of residence*				
LSOA fifth income deprivation quintile	0.022	0.530	0.043	0.038
% LSOA pop non white	0.001	0.461	0.001	0.211
% LSOA not good SAH	$-$0.013	0.020	0.003	0.352
% LSOA households single pensioner	0.004	0.103	$-$0.001	0.334
London LSOA	0.179	0.112	$-$0.126	0.103
LSOA within 10 km of Scottish border	0.860	0.000	$-$0.008	0.899
LSOA within 10 km of Welsh border	0.022	0.765	0.002	0.981
Town and fringe	0.040	0.259	$-$0.039	0.051
Village or hamlet and isolated dwellings	0.007	0.879	$-$0.005	0.833
Population within 10 km (100000 s)	0.044	0.667	0.184	0.000
*Formal long-term care*				
Beds within 10 km second quintile	0.020	0.655	$-$0.026	0.289
Beds within 10 km third quintile	$-$0.052	0.411	$-$0.050	0.153
Beds within 10 km fourth quintile	$-$0.061	0.522	$-$0.111	0.035
Beds within 10 km top quintile	$-$0.196	0.167	$-$0.209	0.003
Price within 10 km second quintile	$-$0.013	0.786	$-$0.024	0.449
Price within 10 km third quintile	$-$0.060	0.247	0.018	0.617
Price within 10 km fourth quintile	$-$0.046	0.530	$-$0.016	0.678
Price within 10 km top quintile	$-$0.051	0.624	$-$0.032	0.477
Care home ratings within 10 km mean	0.122	0.206	$-$0.073	0.144
Constant	3.069	0.000	1.678	0.000
Hospital effects	FE		FE	
$\hbox {R}^{2}$	0.253		0.337	
Observations	4465		28636	

Dependent variable: natural logarithm of length of stay. Coefficients are the proportionate change in length of stay in days from a one unit increase in the explanatory variable. Fixed effects panel data models with cluster robust standard errors. The $\upchi ^{2}$ statistic for the auxiliary regression test (see “Methods” section) for the consistency of the random error specification is $\upchi ^{2}$(37)= 114.35, $p = 0.000$ for patients discharged to care and $\upchi ^{2}$(37)= 450.81, $p = 0.000$ for those discharged to their own home

Greater supply of long-term care beds is not associated with the length of stay of patients discharged to a care home. In contrast, more long-term care beds reduces length of stay for patients discharged home (by 21% in the highest quintile) and the association is significant at 5% in the fourth and fifth quintiles. This is surprising since we would not expect beds supply to affect the hospital length of stay of patients discharged to their own home. Length of stay is not associated with the care home prices whether patients are discharged home or to care home.

### Robustness checks

We tested the sensitivity of our results to different estimation methods and specifications. We interacted income deprivation with the supply and price variables and found that their effect did not vary with deprivation. We measured long-term care supply using patient areas of 20 and 30 km radii instead of 10 km. We estimated models with beds per capita, rather than entering beds and population separately. We estimated Cox proportional hazard models for length of stay. The key results were not affected and are reported in Gaughan et al. (2013).

Table 5 has results from the selection correction model which allows for the interdependence of decisions on length of stay and discharge destination. They are very similar to those from the simpler separate linear models and indicate that accounting for selection does not alter the main findings.

**Table 5:** Comparison of selection correction and separate linear models

	Discharge destination				Ln Length of stay if discharged to care				Ln length of stay if discharged home			
Hip fracture models												
	Linear FE		Selection correction 1st stage		Linear FE		Selectin correction 2nd stage		Linear FE		Selection correction 2nd stage	
	b	p	B	p	b	p	b	P	b	p	b	p
Beds within 10 km 2nd quintile	0.009	0.396	0.042	0.335	$-$0.049	0.336	$-$0.036	0.445	$-$0.012	0.466	$-$0.013	0.426
Beds within 10 km 3rd quintile	0.021	0.088	0.105	0.070	$-$0.064	0.391	$-$0.028	0.669	0.011	0.598	0.012	0.602
Beds within 10 km 4th quintile	0.025	0.263	0.114	0.159	$-$0.216	0.036	$-$0.181	0.046	0.007	0.835	0.008	0.799
Beds within 10 km top quintile	0.041	0.197	0.176	0.141	$-$0.319	0.020	$-$0.246	0.061	$-$0.022	0.641	$-$0.022	0.635
Price within 10 km 2nd quintile	$-$0.010	0.339	$-$0.065	0.173	$-$0.014	0.769	$-$0.024	0.643	$-$0.030	0.178	$-$0.031	0.086
Price within 10 km 3rd quintile	$-$0.012	0.326	$-$0.086	0.143	0.006	0.925	$-$0.010	0.883	$-$0.044	0.116	$-$0.044	0.043
Price within 10 km 4th quintile	0.006	0.739	0.009	0.906	0.162	0.030	0.203	0.015	$-$0.031	0.342	$-$0.030	0.270
Price within 10 km top quintile	$-$0.004	0.817	$-$0.035	0.703	0.175	0.050	0.211	0.040	$-$0.010	0.792	$-$0.009	0.789
Inverse Mills ratio							0.233	0.000			$-$0.324	0.035
*Stroke models*												
	b	p	B	p	b	p	b	P	b	p	b	p
Beds within 10 km 2nd quintile	0.002	0.790	0.019	0.598	0.020	0.655	0.023	0.596	$-$0.026	0.289	$-$0.022	0.346
Beds within 10 km 3rd quintile	$-$0.012	0.238	$-$0.061	0.204	$-$0.052	0.411	$-$0.075	0.213	$-$0.050	0.153	$-$0.065	0.045
Beds within 10 km 4th quintile	0.018	0.188	0.091	0.162	$-$0.061	0.522	$-$0.048	0.551	$-$0.111	0.035	$-$0.092	0.036
Beds within 10 km top quintile	0.014	0.512	0.079	0.410	$-$0.196	0.167	$-$0.174	0.143	$-$0.209	0.003	$-$0.199	0.002
Price within 10 km 2nd quintile	0.002	0.855	0.004	0.929	$-$0.013	0.786	$-$0.004	0.932	$-$0.024	0.449	$-$0.025	0.349
Price within 10 km 3rd quintile	0.011	0.267	0.059	0.206	$-$0.060	0.247	$-$0.032	0.572	0.018	0.617	0.030	0.335
Price within 10 km 4th quintile	0.019	0.127	0.092	0.116	$-$0.046	0.530	$-$0.028	0.696	$-$0.016	0.678	0.003	0.936
Price within 10 km top quintile	0.020	0.175	0.088	0.235	$-$0.051	0.624	$-$0.037	0.698	$-$0.032	0.477	$-$0.014	0.770
Inverse Mills ratio							0.334	0.076			$-$0.778	0.082

There are 21,959 hip fracture observations and 33,101 stroke observations. Linear FE model coefficients are from the models in Tables 2, 3 and 4. Selection correction models include hospital means of patient level variables and deviations from means. Coefficients are those on the deviations

## Discussion

### Discharge destination

As expected, and in line with earlier findings (Bond et al. 2000) older patients and those with greater morbidity are more likely to be discharged to a care home when they leave hospital. Other things equal, men are less likely to enter a care home on discharge. This may be because they are more likely to have a spouse or a partner who can provide informal care at home since women have longer life expectancy and tend to be younger than their partners (Wilson and Smallwood 2008).

There is only very weak evidence in our results that accessibility of long-term care affects the discharge destination for hip fracture or stroke patients. We find that hip fracture patients who live in areas with higher care home beds supply are more likely to be discharged to a care home. The magnitude of the effects are quite large (4.1% points in the highest quintile compared with an unconditional mean probability of 14.5%) but is not statistically significant at the conventional (5%) level.

There is no association between beds supply and discharge destination for stroke patients, who are arguably more severely impaired than hip fracture patients and therefore less able to substitute formal care with informal care at home.

Prices are not associated with discharge destination for either hip fracture or stroke patients. This suggests that the demand for a care home is at most determined by non-monetary costs, such as the waiting time.

In summary, our results suggest that discharge destination is mostly driven by patient characteristics such as severity, age and comorbidities rather than by care home prices and bed supply. Stroke patients discharged to a care home have on average been diagnosed with two additional conditions, received 0.3 more procedures, are more likely to have the more serious haemorrhagic stroke, and to have more comorbidities. Hip fracture patients discharged to care are diagnosed with around one additional condition, receive 0.2 more procedures and have a rate of comorbidity 5 or 6% higher than those discharged home.

### Length of stay

Patient severity is also a key determinant of length of stay. More secondary diagnoses and procedures are associated with significantly longer stays. The primary diagnosis is also important. The effect of age is similar for hip fracture and stroke patients discharged to their own homes: length of stay is over 30% greater for patients over 85. Older hip fracture patients also have longer stays if discharged to care homes, though the effects of age are around one third as large as for patients discharged to their own homes. Counter-intuitively, older stroke patients discharged to a care home have shorter hospital stays. These most complex patients may have the highest priority for nursing home care and have least access to potential substitutes with care at home, a hypothesis supported in part by the significantly longer stay for older patients who are discharged home.

Patients in areas with greater income deprivation who are discharged to their own home stay longer in hospitals. Cookson and Laudicella (2011) also find that English elective hip-replacement patients in poorer areas have greater length of stay. Since poorer individuals are generally in worse health, income deprivation may also be a proxy of poor health and hospitals may want patients discharged home to be in better health than those discharged to a care home where there will be more support.

Within a given hospital, hip fracture patients who are discharged to a care home have a shorter hospital stay if they live in an area with a greater supply of long-term care beds. The results are consistent with bed blocking: once patients are medically fit for discharge their length of stay is determined by factors outside the control of the hospital, such as local care home supply.

We also find that hip fracture patients living in areas with higher long-term care prices have longer hospital stays. This suggests that patients intending to enter a care home take longer to search for a place when bed prices are higher.

The difference between the length of stay of patients discharged home and to a care home is particularly marked for stroke patients. This is expected as stroke is in general a more severe condition. Most critically, the prognosis following a stroke ranges widely from a very short stay of a few days to a protracted stay extending into months. The latter case, involving extensive rehabilitation in a hospital setting, is where patients ultimately discharged to care are concentrated. Patients discharged to their own home quickly are likely to have been less severe and have been treated most promptly as these are important factors in determining outcomes. Stroke patients discharged to a care home are much more likely to have a severe comorbidity (28% compared to 21%) and also more likely to have had a stroke caused by haemorrhage (16% compared to 13%).

The length of stay of stroke patients discharged to a care home is not affected by the availability of beds, their price or the quality of care homes in their area. The differences in results between stroke and hip fracture may be explained by stroke being a more impairing condition for which patients require much more intensive post hospital care. The demand for long-term care for stroke patients may therefore be less affected by beds supply and prices. Note however that the coefficients in the top quintiles are negative and for the top quintile the coefficient is quantitatively large (a reduction in length of stay by 20%) though not statistically significant ($p = 0.17$).

In contrast to hip fracture patients, we find that for stroke patients, greater availability of beds reduces length of stay of those discharged to their own home: patients in the highest quintile of beds supply have a 21% shorter stay than those in the lowest quintile. This may be due to care homes beds supply being positively correlated with provision of assistance for stroke patients in their homes.

As in the case of discharge destination decision, patient severity is the key determinant of length of stay. However, for hip fracture patients the availability of formal long-term care also appears to have an important impact for those discharged to institutional care. For patients discharged to their own home the availability of informal care from relatives is also likely to affect length of stay. We do not have patient-level data on the home circumstances of hospital patients but do use a small area measure of the proportion of pensioners living alone. We find, in line with Picone et al. (2003), that hip fracture patients discharged to their home have longer hospital stays in small areas with a higher proportion of single pensioners.

### Concluding remarks

Our results suggest that accessibility of long-term care matters for hip fracture patients. Greater long-term care supply and lower prices are associated with shorter hospital length of stay for patients discharged to care homes. The effect can be quantitatively large with 20–30% shorter length of stay for patients with most availability (at the highest bed quintiles).

The results are substantially different for stroke patients. Hospital length of stay is not associated with price (in contrast to hip fracture patients). Counter-intuitively, those discharged home have a shorter length of stay if availability of long-term care beds is high. The differences between stroke and hip fracture may be that stroke results in more severe and longer lasting impairment.

Overall, for hip fracture patients we find evidence consistent with the ‘bed-blocking’ hypothesis that availability of long-term care affects the length of stay of patients who no longer need to be in acute hospital and are ready to be discharged. Caring for such patients in hospital is more costly than long-term care. Our results suggest that for hip fracture patients an expansion of the long-term care sector can reduce hospital length of stay and reduce the total cost of caring for these patients.

## Data appendix

A patient is admitted from home if their admission code is “usual place of residence, including no fixed abode” or “temporary place of residence when usually resident elsewhere, for example, hotels and residential educational establishments” (admisorc = 19, 29). Care homes are not considered usual residence and are therefore excluded (see more below). Each HES record covers a single finished consultant episode (FCE) during which the patient is continuously under a single consultant (senior hospital doctor).

We link FCEs into continuous inpatient stays (CIPS) to allow for changes of consultant, including transfers to other hospitals. We combine FCEs into CIPS using the methodology in Castelli et al. (2008) and Cookson and Laudicella (2011). We include patients whose CIPS finish in the financial year 1 April 2008–31 March 2009 and start between 1 April 2007 and 31 March 2009.

Patients are coded as being discharged to their home if their HES discharge destination field indicates usual or temporary residence (disdest 19, 29), to a long-term care facility if their destination is an NHS-run nursing home, a residential home or group home (disdest 54), a local authority care home (disdest 69) or non-NHS (other than local authority) residential care home (disdest 85).

Hip fracture patients have a primary diagnosis ICD10 code of S72.0 (fracture of neck of femur or unspecified femur fracture), S72.1 (pertrochanteric fracture) or S72.2 (subtrochanteric fracture) (Jarman et al. 2004). Stroke patients have primary ICD10 code I60-2 (intracerebral haemorrhage), I63 (cerebral infarction), I64 (unspecified stroke), I66 or I67.2, I69.8 or R47.0 (other form of stroke).

There are 33,082 hip fracture and 59,316 stroke emergency admissions in our study period. We exclude 11,113 hip fracture and 26,211 stroke patients from the analysis because: the patient dies in-hospital (4253 for hip fracture and 15,501 for stroke), the hospital spell is incomplete (2080 for hip fracture and 2518 for stroke), the patient is discharged elsewhere than to usual residence or care home (1595 for hip fracture and 3211 for stroke), is admitted from elsewhere than usual residence where “usual residence” excludes a care home (1910 for hip fracture and 2437 for stroke), has a repeat emergency admission (376 for hip fracture and 1194 for stroke), is treated in a hospital with 10 or fewer cases in 2008/2009 (46 for hip fracture and 60 for stroke). We exclude cases with very long length of stay, and the logarithm of the length of stay is more than three standard deviations above the mean (205 for hip fracture), and cases with missing data (658 for hip fracture and 1294 for stroke).

HES records the patient’s Lower Super Output Area (LSOA) of residence. There are 32,482 LSOAs in England with an average population of 1500 in 2001. We compute the number of beds in care homes within 10 km of the centroid of the patient’s LSOA of residence. For each provider, we have the minimum and maximum price by type of room (single, shared) and type of care (nursing, non-nursing). We compute the average price for care homes within 10 km of each LSOA centroid. 1682 care homes (14%) do not report any price and we impute the price of these from the average price for providers in the same quintile of beds supply. We measure the average quality of care homes within 10 km by assigning numerical values 1–4 to the CQC quality ratings (poor, adequate, good, and excellent). We use the same strategy as for missing price data to impute quality rating for 1953 providers without information (16.5%).

## Footnotes

1 “Care home” is the usual term in England for a residential institution providing long-term personal care such as help with bathing and dressing. Some care homes also provide nursing care and are referred to as nursing homes (Age UK 2015). A care home which does not provide nursing care has, in the past, been sometimes referred to as a residential home. Other countries use a different terminology. For example, in the U.S. care homes might be referred to as Assisted Living, Personal Care Home or Residential Care Facilities with precise definitions determined at the state level. Skilled Nursing Facilities or Nursing Homes perform the same general function as nursing homes in the U.K. (Pioneer Network 2011). As this study uses English data, we use the English terminology definitions and so care homes include nursing and residential homes.

2 The 152 Local Authorities (LAs) with responsibility for social care are elected political bodies funded by local taxes and central government grants. The 166 local acute health trusts are part of the NHS and are appointed by the Department of Health.
